# Renal Clear Cell Carcinoma and Tonsil Metastasis

**DOI:** 10.1155/2013/315157

**Published:** 2013-12-17

**Authors:** Dario Marcotullio, Giannicola Iannella, Gian Franco Macri, Caterina Marinelli, Melissa Zelli, Giuseppe Magliulo

**Affiliations:** ^1^Organi di Senso Department, Sapienza University of Rome, 00165 Rome, Italy; ^2^Otolaryngology Department, University of L' Aquila, Italy

## Abstract

Renal cell carcinoma is the most common renal tumor in adults. Clear cell carcinoma represents 85% of all histological subtypes. In February 2012 a 72-year-old woman came to our department due to the appearance of massive hemoptysis and pharyngodinia. Previously, this patient was diagnosed with a renal cell carcinoma treated with left nephrectomy. We observed an exophytic, grayish, and ulcerated mass in the left tonsillar lodge and decided to subject the patient to an immediate tonsillectomy. Postoperative histology showed nests of cells with highly hyperchromatic nuclei and clear cytoplasm. These features enabled us to make the diagnosis of renal clear cell carcinoma metastasis. Only few authors described metastasis of renal cell carcinoma in this specific site.

## 1. Introduction

Renal cell carcinoma (RCC) is the most common renal tumor in adults. In 85% of cases clear cell carcinoma is the histological subtype. RCC metastasizes mainly to the lung, liver, or bones while head and neck metastases are extremely rare, with possible lesions to the parotid gland, thyroid, paranasal sinuses, and skull [1–3].

In this report we present a rare case of RCC metastasis to the tonsil lodge, which appeared 3 years after left nephrectomy. In the English-language literature only few authors described this site of RCC metastasis [4–8].

## 2. Case Report

In February 2012 a 72-year-old woman came to our department due to the appearance of massive hemoptysis, pharyngodinia, and dysphagia. In 2009, this patient was diagnosed with a renal clear cell carcinoma treated with left nephrectomy. A control CT performed 6 months later showed 2 subpleural pulmonary nodules of about 10 mm in diameter, referable to carcinoma metastases. Therefore, subsequently, the patient was subjected to 6 cycles of chemotherapy treatment.

Pharyngoscopy revealed an exophytic, grayish, and ulcerated mass in the left tonsillar lodge. It measured about 4 cm in maximum diameter and was friable, painful to pressure, and covered with serosanguineous material. No laterocervical lymphadenopathy was evident at neck palpation.

**Figure 1:**
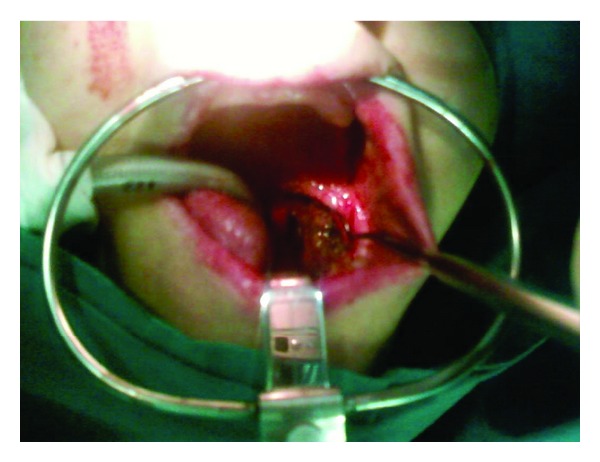
Exophytic, grayish, and ulcerated mass in the left tonsillar lodge (intraoperative image).

Due to the significant bleeding we subjected the patient to immediate surgery. We were able to remove the tonsil mass stopping the bleeding (Figure 1). At intraoperative observation the neoformation did not show infiltration of neighboring structures.

**Figure 2:**
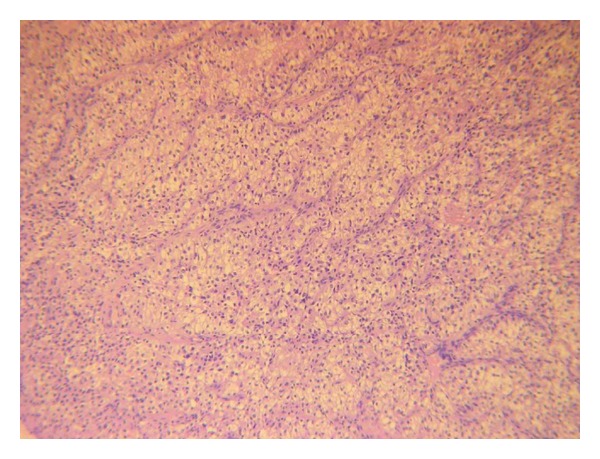
Nests of cells coated with Malpighian epithelium and delimited by fibrous septa. Cells show highly hyperchromatic nuclei and clear cytoplasm (hematoxylin and eosin, 10x).

At postoperative histology nests of cells coated with Malpighian epithelium delimited by fibrous septa were evident. Cells showed highly hyperchromatic nuclei and clear (eosinophilic) cytoplasm (Figure 2). All the above aspects enabled us to make the diagnosis of tonsillar metastasis from renal clear cell carcinoma. A cycle of postoperative radiotherapy was performed. After 6-month followup no recurrence in the head neck region was observed.

## 3. Discussion

RCC represents 3% of all adult malignant tumors and often affects men from the third to sixth decades of life. Clear cell is the most common histological variant of RCC [1–3].

Sites of metastasis are frequently the lung, liver, or bones [1, 3, 9]. Approximately 15% of patients with RCC have extracranial head and neck metastases, with lesions to the parotid gland, dorsal tongue, thyroid, paranasal sinuses, and skull [1, 3].

Metastases in the tonsil lodge have been reported previously only in five cases [4–8]. In our patient the initial appearance of hemoptysis and dysphagia was interesting.

The clear cell carcinoma consists of rounded or polygonal cells with abundant clear cytoplasm, which contains cholesterol and glycogen. Most of these tumors are well differentiated, but some show characters of cellular atypia. Approximately 50% of RCC specimens express vimentin positivity in immunohistochemical stains [3, 8, 9].

RCC seems to metastasize in 3 ways, either through lymphatic spread, through hematogenous spread, or by Batson's venous plexus. This latter is a paraspinal venous plexus through which tumor emboli bypass the normal lung filtration system, producing metastasis to the head and neck region without lung involvement [10].

CT scan is the radiologic investigation of choice in assessing the extent of the metastatic lesion, particularly to the head and neck. MRI can also be helpful, especially in assessing residual disease after treatment [10, 11].

The treatment of choice for RCC is nephrectomy. The best treatment for the head and neck metastases has not yet been clearly established; nevertheless it should be chosen according to the affected site and to the patient's general health [12]. Surgery is recommended as the primary line of treatment especially for those with no other organ involvement [3]. We opted for this type of intervention mainly to stop the bleeding originating from the tonsillar lodge. Moreover in our case the surgery reduced pain and dysphagia and prevented future infections.

RCC is traditionally described as a radioresistant tumor; in fact the role of radiotherapy as the primary approach is controversial and has been reported only for palliative management [12]. Massaccessi et al. [8] reported in 2009 a case of clear cell renal carcinoma with tonsillar metastases treated only with radiotherapy due to patient's condition. Only a partial reduction of the tumor size to high doses of radiation therapy was observed.

The 5-year survival in patients with head and neck metastases has been reported to be between 0% and 20% [9]. It is also known that favorable prognosis is associated with solitary metastatic focus and longer interval between the primary treatments and the metastasis appearance [13]. In our patient after six months of followup there was no progression of lung disease or new metastasis in the head and neck region.

In conclusion, the possibility of metastasis from clear cell RCC in cases of tonsillar lodge masses should be considered. A prompt surgical intervention, in case of bleeding, is strongly recommended.

## References

[1] Demir L, Erten C, Somali I (2012). Metastases of renal cell carcinoma to the larynx and thyroid: two case reports on metastasis developing years after nephrectomy. *Canadian Urological Association Journal*.

[2] Lee HM, Kang HJ, Lee SH (2005). Metastatic renal cell carcinoma presenting as epistaxis. *European Archives of Oto-Rhino-Laryngology*.

[3] Pritchyk KM, Schiff BA, Newkirk KA, Krowiak E, Deeb ZE (2002). Metastatic renal cell carcinoma to the head and neck. *Laryngoscope*.

[4] Stańczyk R, Omulecka A, Pajor A (2006). A case of renal clear cell carcinoma metastasis to the oropharynx. *Otolaryngologia Polska*.

[5] García Lozano MC, Fernández Gómez J, Lloret Selles E, Delgado Quero A, Galdeano Granda E (1998). Metastasizing hypernephroma in palatine tonsil. *Anales Otorrinolaringologicos Ibero-Americanos*.

[6] Menauer F, Issing WJ (1998). Unusual metastasis of a hypernephroma. Case report and literature review. *Laryngo-Rhino-Otologie*.

[7] Green KMJ, Pantelides E, de Carpentier JP (1997). Tonsillar metastasis from a renal cell carcinoma presenting as a quinsy. *Journal of Laryngology and Otology*.

[8] Massaccesi M, Morganti AG, Serafini G (2009). Late tonsil metastases from renal cell cancer: a case report. *Tumori*.

[9] Yoskovitch A, Nguyen LHP, Sadeghi N, Auger M (2001). Renal cell carcinoma presenting as a mandibular mass. *Otolaryngology—Head and Neck Surgery*.

[10] Gottlieb MD, Roland JT (1998). Paradoxical spread of renal cell carcinoma to the head and neck. *Laryngoscope*.

[11] Gil-Julio H, Vázquez-Alonso F, Fernández-Sánchez AJ, Puche-Sanz I, Flores-Martín JF, Cózar JM (2012). Metastasis of renal cell carcinoma to the buccal mucosa 19 years after radical nephrectomy. *Case Reports in Oncological Medicine*.

[12] Marioni G, Gaio E, Poletti A, Derosas F, Staffieri A (2004). Uncommon metastatic site of renal adenocarcinoma: the oral tongue. *Acta Oto-Laryngologica*.

[13] Moudouni SM, Tligui M, Doublet JD, Haab F, Gattegno B, Thibault P (2006). Late metastasis of renal cell carcinoma to the submaxillary gland 10 years after radical nephrectomy. *International Journal of Urology*.

